# Enhancement of plant growth in lentil (*Lens culinaris*) under salinity stress by exogenous application or seed priming with salicylic acid and hydrogen peroxide

**DOI:** 10.1371/journal.pone.0326093

**Published:** 2025-06-20

**Authors:** Amal Bouallegue, Faouzi Horchani, Fatma Souissi, Mohamed Tebini, Karima Jalali, Hela Ben Ahmed, Zouhaier Abbes, Haythem Mhadhbi

**Affiliations:** 1 Laboratory of Legumes and Sustainable Agrosystems, Biotechnology Center of Borj Cedria, Hammam-Lif, Tunisia; 2 Laboratory of Biotechnology and Biomonitoring of the Environment and Oasis Ecosystems, Gafsa University, Faculty of Sciences of Gafsa, Zarroug University of Gafsa, Gafsa, Tunisia; 3 Plant-Soil-Environment Interactions Laboratory, Faculty of Sciences of Tunis, University of Tunis EL Manar, Tunis, Tunisia; 4 Field Crop Laboratory, National Institute for Agricultural Research of Tunisia, University of Carthage, Tunis, Tunisia; Graphic Era Institute of Technology: Graphic Era Deemed to be University, INDIA

## Abstract

This study was conducted in order to test the effect of seed pretreatment or exogenous application through the rooting medium of 0.1 mM Salicylic Acid (SA) and 0.1 mM hydrogen peroxide (H_2_O_2_) on growth, nutritional behavior and some biochemical parameters (photosynthetic pigments, gas exchange parameters, oxidative stress indicators and antioxidant enzymes activities) of lentil plants (*Lens culinaris*) under 75 mM salt stress. Our results demonstrated that salt stress noticeably reduced shoot and root DWs by 39.01 and 42.81%, respectively, as compared to controls. This reduction was associated with a significant decrease in all photosynthetic parameters, including Chlorophyll (Chl) and carotenoid (Car), net assimilation of photosynthesis (*A*), stomatal conductance (*gs*), transpiration (*E*) and internal CO_2_ level (*Ci*), an accumulation of Na^+^ and Cl^-^ and a decrease of K^+^ and Ca^2+^ concentrations in plant shoots and roots. In addition, relative to control plants, salt stress remarkably increased the malondialdehyde MDA and H_2_O_2_ contents especially in roots and increased GPOX and SOD activities, especially in plant shoots. Both methods of SA and H_2_O_2_ application recovered the plant growth, enhanced shoot and root DWs (increase of 67.65 and 82.36% in shoots and roots, respectively, as compared to salt-stressed plants) and increased all parameters that were reduced by NaCl treatment. Nevertheless, the most prominent effects of SA and H_2_O_2_ on plant growth were obtained with the seed priming method. Thus, SA and H_2_O_2_ applications, especially the H_2_O_2_ seed priming method, induced the antioxidant system, improved the membrane stability and ameliorated the gas exchange parameters. As compared to salt plant stressed, Na^+^ and Cl^-^ contents were significantly decreased and K^+^ and Ca^2+^ were significantly increased in shoots and roots following SA and H_2_O_2_ applications, especially with the H_2_O_2_ seed priming method. Similarly, this method was more efficient in alleviating the adverse effects of salt stress on all photosynthetic pigment contents and measured gas exchange parameters. Compared to salt stressed plants, it significantly decreased the H_2_O_2_ and MDA contents and further stimulated GPOX and SOD activities. Our results indicated that the seed priming method, particularly with H_2_O_2_, could be recommended for obtaining better growth of lentil seedlings under salt-affected soil conditions.

## Introduction

Salt stress is considered as an abiotic stress that severely affects crop production, especially in arid and semi-arid areas, due to scarcity of rains and increased evapotranspiration [1]. Salt stress affected, approximately, 33% of irrigated crop lands, and by 2050, this could exceed 50% [2].

Salt stress disturbs growth and developmental processes by influencing several physiological and biochemical plant’s processes [3]. These influences can occur in two phases; the first one is related to the high salt concentration in root zones (osmotic effect), whereas the second is related to high accumulation of salt ions in plant tissues (toxic effect) [4]. Moreover, it is well established that plants exposed to salt stress showed a production of high amounts of reactive oxygen species (ROS) resulting thereby in the deactivation of various important cellular and metabolic processes in plants [3,5]. In line with this, several studies showed that salt stress results in alteration in the ultra-structural cell components, decrease in the amount of chlorophyll pigments and in enzymatic activities, disturbance of the stomatal conductance and the photosynthesis machinery limiting thereby the development and productivity of crops [3,6].

Lentils (*Lens culinaris*) are considered as one of the most important legumes due to their numerous benefits [7,8]. In addition to their high-quality source of vitamins, micronutrients, dietary fibers, oligosaccharides, fatty acids and vitamins, lentils are considered as an essential crop due to their high protein contents [9–11]. Despite their ability to grow and produce in some marginal environments, such as extremely cold temperatures and drought, lentils are generally vulnerable to salt stress during all plant’s life stages, which may reduce their yield up to 50% [12].

Nowadays, to alleviate water scarcity, the irrigation of crop plants with saline water has become necessary, especially in semi-arid and arid areas. Thus, it is extremely important to identify new strategies that minimize the deleterious effects of the use of saline waters on plants [13]. An alternative strategy that can be used is the application of growth regulators, fertilizers, osmoprotectants and antioxidants [14–17]. During the last two decades, salicylic acid (SA) has been the focus of intensive research as a plant growth regulator with various roles in many plants’ biological processes [18,19]. Besides its important role in plant growth and development, SA has emerged as secondary metabolite involved in the regulation of respiration, photosynthesis and enzyme biosynthesis [19]. Furthermore, several previous studies showed that SA acts in the regulation of the plant resistance response to different abiotic stresses, particularly salt stress [20,21]. The ability of SA to mitigate the adverse effects of salt stress has been demonstrated in many crops such as cabbage [22], cowpea [23], strawberries [24] and alfalfa [25]. As already reported, this SA-mediated tolerance can be achieved through the regulation of a wide range of vital physiological and biochemical processes such as water relations, stomatal conductance, photosynthesis, ion homeostasis, cell membrane permeability as well as the regulation of the osmotic adjustment system and the activation of the antioxidant enzymes to counter the deleterious effects of ROS [19,26].

On another hand, H_2_O_2_, as a signaling molecule, plays an important role in plant developmental processes [27–29]. Several previous studies showed that H_2_O_2_ is involved in the regulation of various biological processes, such as increase of Ca^2+^ concentration in plants and the synthesis of osmolytes such as proline, resulting in osmotic adjustment [30,31]. Furthermore, apart from its involvement in the general plant’s growth and metabolism, H_2_O_2_ is involved in the modulation of many fundamental functions in plants under marginal soil conditions, mainly salt-affected soils [28,32,33]. For instance, it has been shown that exogenous application of H_2_O_2_ alleviated the negative effects of salt stress on photosynthesis and production components in bell pepper plants [30]. Recently, Nobrega et al. [33] showed that the application of H_2_O_2_ reduced the effects of salt stress on several photosynthesis parameters of cotton plants under salt stress.

As described above, several studies and arguments demonstrated the ability of SA and H_2_O_2_ to regulate the plant tolerance-responses to salt stress. Nevertheless, the SA and H_2_O_2_-mediated tolerance depends on several parameters like the salt stress concentration, duration of stress application, plant species, the plant growth stage as well as the method of application of these two chemical agents and the applied dose [19]. Despite SA and H_2_O_2_ may be applied to plants as seed priming or through exogenous supplementation, data regarding to the efficiency of these methods under salt stress conditions are scarce. Therefore, this study was conducted in order to evaluate the effect of seed pretreatment or exogenous application through the rooting medium of SA and H_2_O_2_ on growth, mineral nutrition, photosynthetic pigments, gas exchange parameters, oxidative stress indicators and antioxidant enzymes’ activities of lentil plants under salt stress. This is one of the few works released to compare the effectiveness of exogenous application or seed pretreatment with SA and H_2_O_2_ on salt stressed plants, especially on *Lens culinaris*.

## Materials and methods

### Growth conditions and experimental designs

Seeds of lentil cultivar Ncir were sterilized by soaking in 3.6% sodium hypochlorite solution, followed by washing with sterile distilled water. In a first set of experiment, seeds were unprimed (control) or primed by soaking for 3 hours in 0.1 mM salicylic acid (SA) or 0.1 mM hydrogen peroxide (H_2_O_2_). Unprimed and primed seeds were then germinated in Petri plates (15 seeds per Petri plate) double lined with moistened filter paper in the dark at 25°C. Ten days after germination, seedlings obtained from unprimed and primed seeds were grown hydroponically in a glasshouse at 26°C/20°C day/night temperature, 70–80% relative humidity, a 16 h photoperiod and using a nutrient solution with the following composition: KH_2_ PO_4_ (0.36 mM), K_2_ SO_4_ (0.7 mM), MgSO_4_,7H_2_O (1 mM), CaCl_2_, 2H_2_O (1.65 mM), Urée (4 mM), Sequestrène (1.26 mM), MnSO_4_,H_2_O (6.6 mM), ZnSO_4_,7H_2_O (1.55 mM), CuSO_4_,7H_2_O (1.56 mM), H_3_BO_3_ (4 mM), CaSO_4_,7H_2_O (0.12 mM), Na_2_MoO_4_ (0.12 mM) [34]. Salt treatment was applied 30 days after seedling transplantation by adding 75 mM NaCl in the culture medium. The concentrations of 75 mM NaCl-salinity, 0.1 mM SA and 0.1 mM H_2_O_2_ used in this work were selected on the basis of previous published experiments [28,32,35–37]. In a second set of experiment, SA (0.1 mM), H_2_O_2_ (0.1 mM) and NaCl (75 mM) were exogenously applied through the rooting medium to thirty-day-old seedlings obtained from unprimed seeds. The experiment was conducted in a randomized complete block design with three replications. Treatment conditions followed in the two sets of experiments are shown below.

Denotation of the used treatment

**Table:** 

Treatment	Denotation
Control (without NaCl and chemical agent treatments)	Control
75 mM NaCl-salinity (without chemical agent treatment)	NaCl
Seeds primed with 0.1 mM SA (without NaCl treatment)	SA/SP
Seeds primed with 0.1 mM H_2_O_2_ (without NaCl treatment)	H_2_O_2_/SP
Exogenous application of 0.1 mM SA (without NaCl treatment)	SA/EA
Exogenous application of 0.1 mM H_2_O_2_ (without NaCl treatment)	H_2_O_2_/EA
Seeds primed with 0.1 mM SA (with 75 mM NaCl treatment)	NaCl + SA/SP
Seeds primed with 0.1 mM H_2_O_2_ (with 75 mM NaCl treatment)	NaCl + H_2_O_2_/SP
Exogenous application of 0.1 mM SA (with 75 mM NaCl treatment)	NaCl + SA/EA
Exogenous application of 0.1 mM H_2_O_2_ (with 75 mM NaCl treatment)	NaCl + H_2_O_2_/EA

### Plant harvest and dry weight determination

Thirty days after treatment applications, roots and shoots were harvested and fresh and dry weights were immediately determined. Dry weights were obtained after drying plant material at 80°C for 3 days.

### Mineral analysis

Dried tissues were digested with 7% nitric acid (HNO_3_) for mineral analysis. Na^+^, K^+^ and Ca^2+^ ions were analyzed using a spectrophotometer (Eppendorf Geratebau Netherler). However, Cl^-^ ions were quantified using a Chloridometer (Haake, Buchler instruments Inc., New Jersey, USA).

### Gas-exchange parameters measurement and photosynthetic pigment contents

Measurements of gas-exchange parameters (net CO_2_ rate (*A*), stomatal conductance (*gs*), transpiration rate (*E*) and intern CO_2_ content (*Ci*)) were determined at the end of the experiment using a portable gas exchange system (LCPro + , Bio-Scientific, Great Amwell, Herts, UK). Measurements were released on mature leaves cultivated at 1200 μmol m^-2^ s^-1^ PAR, 400 μmol mol^-1^ CO_2_ concentrations and 29 ± 2°C leaf temperature. Measurements were done on acclimated leaves (15 min in leaf chamber) between 10:00 am and 2:00 pm.

The leaf chlorophyll and carotenoid concentrations were determined and estimated following the methods of Arnon [38] and McKinney [39], respectively. Extracts were prepared using 100 mg FW in 80% chilled acetone.

### Determination of H_2_O_2_ and malondialdehyde MDA contents

The method of Velikova et al. [40] was used to determine the H_2_O_2_ content. Extract absorbance was read at 390 nm. A standard curve of H_2_O_2_ was used in order to estimate contents.

The thiobarbioturic acid (TBA) method [41] was used for lipid peroxidation estimation. Extracts absorbances were recorded at 532 nm (specific) and 600 nm (non-specific).

### Determination of protein content and antioxidant enzymes activities

Tissue samples were homogenized in 50 mM KH_2_PO_4_/ K_2_HPO_4_, pH7.8, and then centrifuged for 15 at 12.000g min at 4°C. The Bradford method [42] was used in order to estimate the tissue protein concentration. Guaiacol peroxidase GPOX activity was measured at 470 nm according to Anderson et al. [43]. However, Superoxide dismutase SOD activity was evaluated at 560 nm according to Lee et al. [44].

### Statistical analysis

Differences between treatments for all measurements were analyzed using the SPSS software (Version 15.0 for Windows). Comparison was carried out at *P *= 0.05 using Duncan’s multiple-range test. All measurements were carried out in triplicate.

## Results

### Shoot and root dry weights under salt stress and different SA and H_2_O_2_ treatments

**Fig 1:**
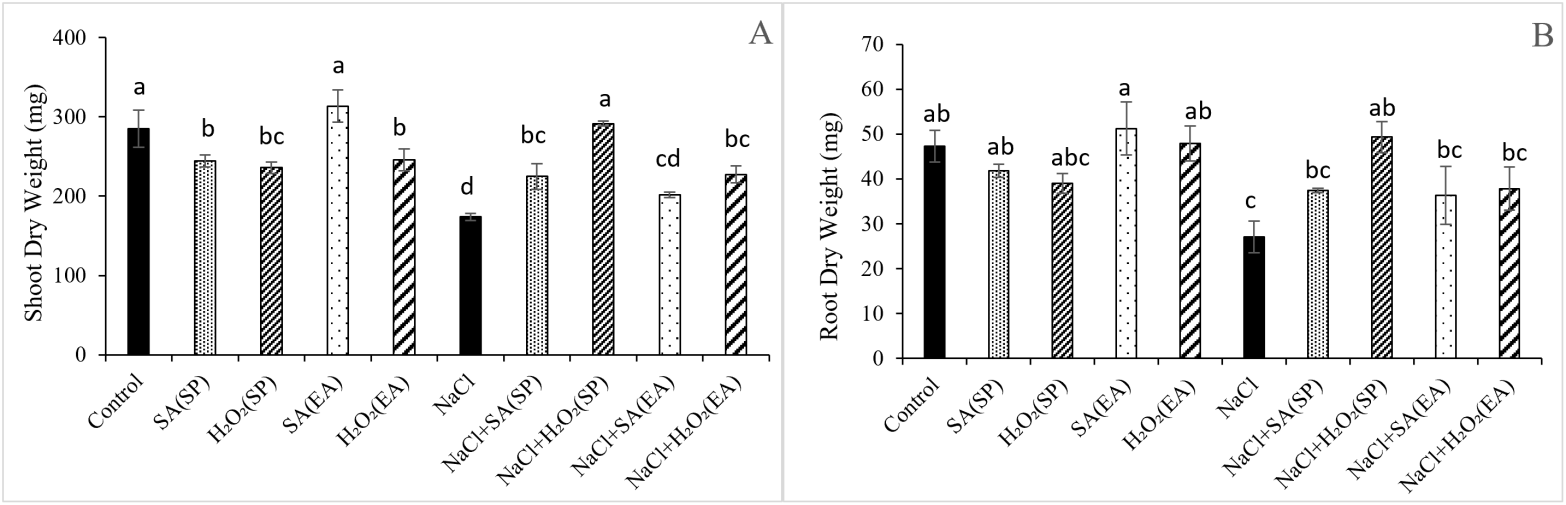
Effect of 0.1mM salicylic acid (SA) or hydrogen peroxide (H_2_O_2_) and salt treatment (0 and 75 mM NaCl) on shoots (A) and roots (B) DW of lentil plants. SA and H_2_O_2_ are applied either by seed’s priming (SP) or added in the culture medium (EA). *Bars are means ± SE. Values with the same letter per parameter are not significantly different according to Duncan test (p = 0.05), n = 3.*

The impacts of SA and H_2_O_2_, applied either through the rooting medium or seed priming, on the DW production of lentil plants under salt stress are displayed in Fig 1. Results showed that salt stress significantly reduced shoot and root DWs by 39.01 and 42.81%, respectively, as compared to controls. Nevertheless, independently of their method of application, SA and H_2_O_2_ treatments alleviated the decrease in the growth of the salt-stressed plants. With the SA and H_2_O_2-_priming method, shoot and root DWs were increased by 29.34% and 38.37 as well as by 67.65% and 82.36%, respectively, compared to plants treated only with NaCl. Similarly, shoot and root DWs were increased by 12.24 and 30.85% as well as 34.03% and 39.92% respectively, following exogenous application through the rooting medium of SA and H_2_O_2_. It is noteworthy that, as compared to the exogenous application, the seed priming method was more efficient in alleviating the negative effects of salt stress on dry matter production of lentil plants, particularly with H_2_O_2_ (increase of 67.65 and 82.36% in shoots and roots, respectively, as compared to salt-stressed plants). Under non saline conditions, SA and H_2_O_2_, applied either through seed priming or the rooting medium, did not affect root DW. However, a slight decrease was observed in shoot DW of plants obtained from SA and H_2_O_2_-primed seeds, relative to unprimed ones (Fig 1).

### Mineral nutrition in lentil plants under salt stress and different SA and H_2_O_2_ treatments

As expected, salt treatment increased Na^+^ and Cl^-^ and decreased the K^+^ and Ca^2+^ concentrations in shoots and roots of lentil plants (Table 1). Although SA and H_2_O_2_, applied either through seed priming or rooting medium, noticeably decreased shoot and root salt ions, obtained results showed that K^+^ and Ca^2+^ concentrations depended on the plant organ and on the used chemical agent as well as its manner of application. As for root Ca^2+^, shoot K^+^ concentration was significantly increased by the both methods of H_2_O_2_ treatment. However, no obvious effects were observed following SA treatment, whatever its method of application. In roots, K^+^ concentration was significantly increased following SA and H_2_O_2_ treatments, independently of their method of application. In salt-stressed lentil plants obtained from seeds primed with SA or H_2_O_2_, shoot Ca^2+^ concentration was increased by 22.09 and 30.33%, respectively, relative to plants treated only by NaCl. Contrarily to the exogenous application of H_2_O_2_, for which we noted an increase of 16.47%, no obvious effects were observed in shoot Ca^2+^ concentration when SA was applied through the rooting medium. Furthermore, among all applied treatments, the most prominent effect on root and shoot K^+^ and Ca^2+^ was obtained with the seed priming method, especially with H_2_O_2_ (Table 1). Under non-saline conditions, SA applied either through the rooting medium or seed priming, significantly increased K^+^ and Ca^2+^ contents, in shoots and roots. However, no obvious effects were observed following treatments with H_2_O_2_, irrespective of its method of application (Table 1).

**Table 1:** Effect of 0.1mM salicylic acid (SA) or hydrogen peroxide (H_2_O_2_) and salt treatment (0 and 75 mM NaCl) on Na^+^, K^+^, Ca^2+^ and Cl^-^ levels in meq. g^-1^DW in shoots and roots of lentil plants. SA and H_2_O_2_ are applied either by seed’s priming (SP) or added in the culture medium (EA).

	Shoots				Roots			
**Na** ^**+**^	**K** ^**+**^	**Ca** ^**2+**^	**Cl** ^**-**^	**Na** ^**+**^	**K** ^**+**^	**Ca** ^**2+**^	**Cl** ^**-**^
**Control**	1,07 ef*	21,14 d	4,60 ab	0,65 d	1,12 de	21,14 d	4,37 a	0,47 d
**SA (SP)**	0,99 f	32,23 a	3,36 cd	0,70 d	1,15 de	27,45 a	3,37 bc	0,37 e
**H**_**2**_**O**_**2**_ **(SP)**	0,97 f	25,20 c	4,72 a	0,68 d	1,14 de	24,50 c	4,52 a	0,38 e
**SA (EA)**	1,02 ef	21,42 d	3,07 de	0,66 d	1,14 de	19,47 e	3,73 b	0,46 d
**H**_**2**_**O**_**2**_ **(EA)**	1,20 e	27,45 b	4,39 b	0,66 d	1,07 e	26,12 b	4,50 a	0,42 de
**NaCl**	2,39 a	13.71 g	2,67 f	1,41 a	1,60 a	6,05 h	2,97 c	0,85 a
**NaCl + SA(SP)**	1,66 c	14,53 g	3,26 cd	1,29 b	1,23 cd	7,52 g	3,41 bc	0,65 c
**NaCl + H**_**2**_ **O**_**2**_ **(SP)**	2,00 b	17.53 e	3,48 c	1,20 c	1,35 b	8,25 fg	3,82 b	0,75 b
**NaCl + SA(EA)**	1,91 b	14,15 g	2,84 ef	1,19 c	1,35 b	7,50 g	3,41 bc	0,68 bc
**NaCl + H**_**2**_ **O**_**2**_ **(EA)**	1,46 d	16,03 f	3,11 de	1,17 c	1,32 bc	8,97 f	3,81 b	0,63 c

*Values with the same letter per parameter are not significantly different according to Duncan test (p = 0.05), n = 3.*

### Gas exchange parameters in lentil plants under salt stress and different SA and H_2_O_2_ treatments

Salt stress significantly decreased all photosynthetic parameters of lentil plants (Table 2). As compared to controls, net assimilation of photosynthesis (*A*), stomatal conductance (*gs*), transpiration (*E*) and internal CO_2_ level (*Ci*), were reduced by 59.58, 83.33, 78.19 and 63.07%, respectively. Nevertheless, all analyzed photosynthetic parameters were noticeably enhanced by SA and H_2_O_2_, irrespective of their method of application. In fact, *A*, *gs*, *E* and *Ci* were increased by 114.23, 266.66, 195.65% and 152.79% as well as 105.65, 66.66, 17.39 and 247.44%, in salt-stressed plants obtained from seeds primed with SA and H_2_O_2_, respectively, as compared to those from unprimed seeds. Similarly, *A*, *gs*, *E* and *Ci* were enhanced by 51.4, 166.66, 106.52 and 111.1% as well as 54.56, 66.66, 43.47 and 209.49%, in salt-stressed plants exogenously treated with SA and H_2_O_2_, respectively. It is noteworthy that the most prominent effects on *A*, *gs*, *E* and *Ci* of salt-stressed plants were obtained following the seed priming method, especially with H_2_O_2_. With some exceptions, SA and H_2_O_2_ did not affect all measured photosynthetic parameters, under non-saline conditions (Table 2).

**Table 2:** Effect of 0.1mM salicylic acid (SA) or hydrogen peroxide (H_2_O_2_) and salt treatment (0 and 75 mM NaCl) on photosynthetic assimilation (*A*), stomacal conductance (*gs*), transpiration (*E*) and intern CO_2_ content (*Ci*) in lentil leaves. SA and H_2_O_2_ are applied either by seed’s priming (SP) or added in the culture medium (EA).

	*A* (µmol CO_2_.m^-2^.s^-1^)	*Gs* (mmol H_2_O.m^-2^.s^-1^)	*E* (mmol H_2_O.m^-2^.s^-1^)	*Ci* (µmol.mol^-1^)
**Control**	13,56 a	0,18 a	2,11 a	371,00 cd
**SA (SP)**	10,05 c	0,16 b	1,32 c	416,33bc
**H**_**2**_**O**_**2**_ **(SP)**	13,47 a	0,15 c	1,43 c	331,67 de
**SA(EA)**	14,65 a	0,18 a	1,86 b	369,67 cd
**H**_**2**_**O**_**2**_ **(EA)**	9,99 c	0,14 c	1,34 c	451,67 ab
**NaCl**	5,48 e	0,03 g	0,46 f	137,00 f
**NaCl + SA(SP)**	11,74 b	0,11 d	1,36 c	346,33 d
**NaCl + H**_**2**_**O**_**2**_ **(SP)**	11,27bc	0,05 f	0,54ef	476,00 a
**NaCl + SA(EA)**	8,30 d	0,08 e	0,95 d	289,33 e
**NaCl + H**_**2**_**O**_**2**_ **(EA)**	8,47 d	0,05 f	0,66 e	424,00 b

Values with the same letter per parameter are not significantly different according to Duncan test (p = 0.05), n = 3

### Photosynthetic pigment contents in lentil plants under salt stress and different SA and H_2_O_2_ treatments

Data regarding photosynthetic pigment contents in salt-stressed lentil plants following treatments with SA and H_2_O_2_ are shown in Table 3. Obtained findings showed that Chl a and Chl b as well as Chl (a + b) and carotenoids were reduced by 27.59, 22.32, 20.81 and 45.38%, respectively in salt-stressed as compared to control plants. Contrarily to Ch (a + b) for which a significant increase was observed only following the H_2_O_2_ primed seeds treatment, Ch a content of salt-stressed plants was noticeably increased by 24.97, 36.77, 26.2 and 22.7% following the application of SA and H_2_O_2_ as seed priming and through the rooting medium, respectively. Except the H_2_O_2_ primed seeds treatment for which a significant increase was observed, all other treatments did not affect Chl b contents. Carotenoid content of salt-stressed plants was increased by 46.24, 32.51 and 29.57%, following the application of SA as seed priming and following the application of SA and H_2_O_2_ either as seed priming or through the rooting medium, respectively; whereas no obvious effect was observed with the H_2_O_2_ primed seeds treatment (Table 3).

**Table 3:** Effect of 0.1mM salicylic acid (SA) or hydrogen peroxide (H_2_O_2_) and salt treatment (0 and 75 mM NaCl) on Chlorophyll a (Chla), Chlorophyll B (Chlb), Carotenoids and Chlorophyll a and b (Chla+b) content in µg.g-^1^ FW in shoots of lentil plants. SA and H_2_O_2_ are applied either by seed’s priming (SP) or added in the culture medium (EA).

	Chla	Chlb	Carotenoids	Chla+b
**Control**	366,56 ab	168,72 abc	15,60 b	535,28 ab
**SA (SP)**	310,82 d	143,25 cd	18,30 a	454,08 c
**H**_**2**_**O**_**2**_ **(SP)**	315,02 d	147,18 cd	13,75bc	462,20 c
**SA (EA)**	384,46 a	178,07 ab	13,80bc	562,53 a
**H**_**2**_**O**_**2**_ **(EA)**	302,28 d	145,31 cd	13,85bc	447,59 c
**NaCl**	265,40 e	131,05 d	8,52 e	423,86 c
**NaCl + SA(SP)**	331,69bcd	151,93bcd	12,46 cd	483,62bc
**NaCl + H**_**2**_**O**_**2**_ **(SP)**	363,00 abc	182,22 a	8,70 e	545,22 b
**NaCl + SA(EA)**	334,96bcd	158,46abcd	11,29 d	466,01 c
**NaCl + H**_**2**_**O**_**2**_ **(EA)**	325,66 cd	156,28abcd	11,04 d	481,94bc

Values with the same letter per parameter are not significantly different according to Duncan test (p = 0.05), n = 3

### H_2_O_2_ and MDA contents in lentil plants under salt stress and different SA and H_2_O_2_ treatments

**Fig 2:**
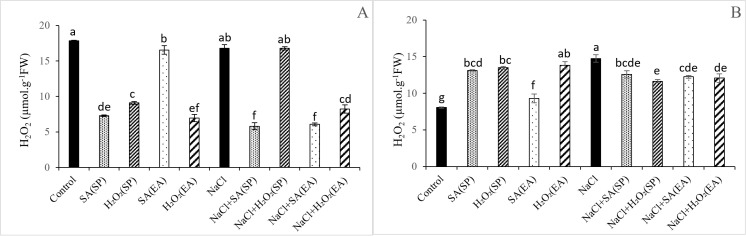
Effect of 0.1mM salicylic acid (SA) or hydrogen peroxide (H_2_O_2_) and salt treatment (0 and 75 mM NaCl) on hydrogen peroxide (H_2_O_2_) content in µmol.g^-1^ FW in shoots (A) and roots (B) of lentil plants. SA and H_2_O_2_ are applied either by seed’s priming (SP) or added in the culture medium (EA). *Bars are means ± SE. Values with the same letter per parameter are not significantly different according to Duncan test (p = 0.05), n = 3.*

**Fig 3:**
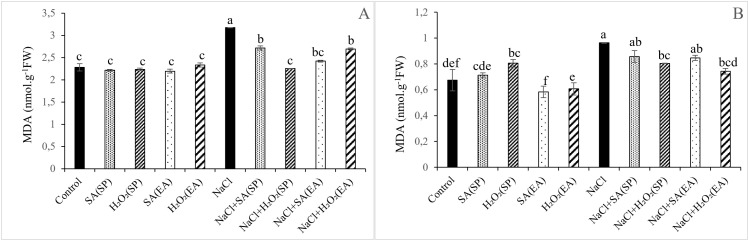
Effect of 0.1mM salicylic acid (SA) or hydrogen peroxide (H_2_O_2_) and salt treatment (0 and 75 mM NaCl) on malondialdehyde (MDA) level in shoots (A) and roots (B) of Lentil plants. SA and H_2_O_2_ are applied either by seed’s priming (SP) or added in the culture medium (EA). *Bars are means ± SE. Values with the same letter per parameter are not significantly different according to Duncan test (p = 0.05), n = 3.*

Results relative to MDA and H_2_O_2_ contents, as oxidative damage markers, are shown in Figs 2 and 3. Salt stress induced the generation of H_2_O_2_ in roots of lentil plants by 83.10%, causing an increase of 42.73% in lipid peroxidation. Although no obvious effect of salt stress was observed in shoot H_2_O_2_ content_,_ MDA concentration was increased by 39.64%, as compared to control. Whatever its method of application, SA was efficient in decreasing the concentrations of H_2_O_2_ both in shoots in roots of salt-stressed plants (Fig 2). Interestingly, MDA contents were significantly decreased in shoots and remained unchanged in roots. It is noteworthy that exogenous application of H_2_O_2_ to salt-stressed plants significantly decreased shoots and roots H_2_O_2_ contents. However, with the seed priming method, H_2_O_2_ content was significantly reduced in roots and remained unchanged in shoots, as compared to plants treated only with NaCl. Concerning lipid peroxidation, our findings showed that MDA contents were noticeably decreased in salt-stressed plants following SA and H_2_O_2_ treatments, independently of their method of application. In roots, MDA contents were significantly decreased by exogenous application and seeds priming with H_2_O_2_. Nevertheless, SA had no obvious effects on root MDA contents (Fig 3). Under non-saline conditions, H_2_O_2_ contents were significantly decreased and increased in shoots and roots, respectively, following SA and H_2_O_2_ treatments, independently of their method of application. Except in roots under the seed priming method, the MDA contents were unaffected by SA and H_2_O_2_ treatments both in roots and shoots (Fig 3).

### Activities of enzymatic antioxidant of lentil plants under salt stress and different SA and H_2_O_2_ treatments

**Fig 4:**
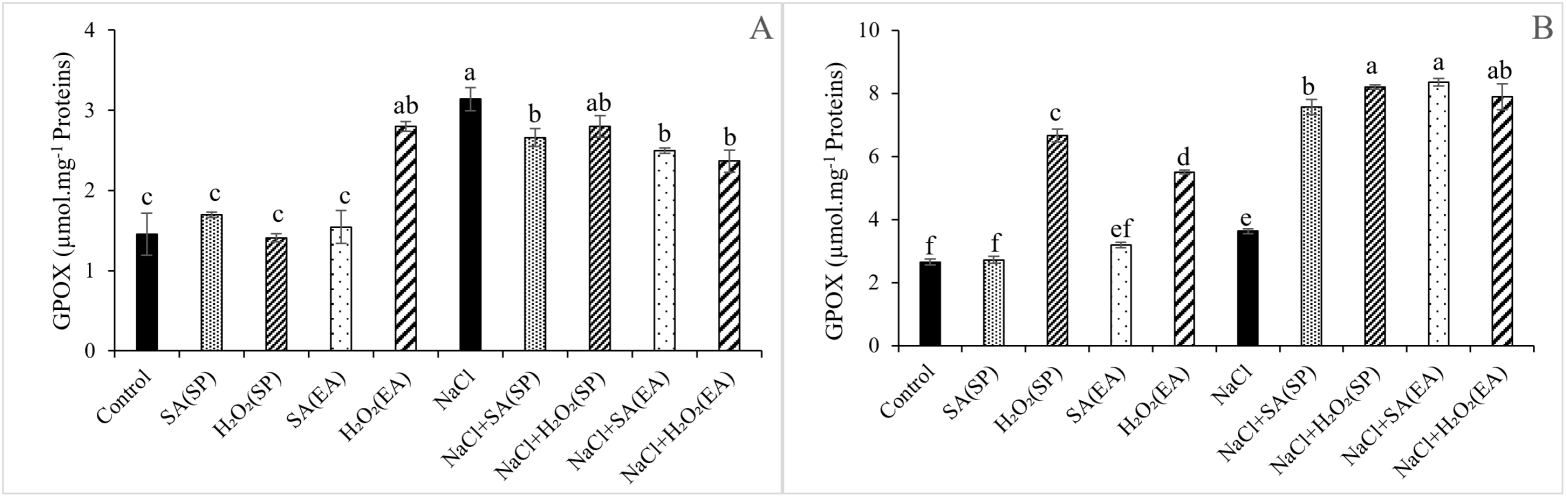
Effect of 0.1mM salicylic acid (SA) or hydrogen peroxide (H_2_O_2_) and salt treatment (0 and 75 mM NaCl) on in the guaicaolperoxydase activity (GPOX) in shoots (A) and roots (B) of lentil plants. SA and H_2_O_2_ are applied either by seed’s priming (SP) or added in the culture medium (EA). *Bars are means ± SE. Values with the same letter per parameter are not significantly different according to Duncan test (p = 0.05), n = 3.*

**Fig 5:**
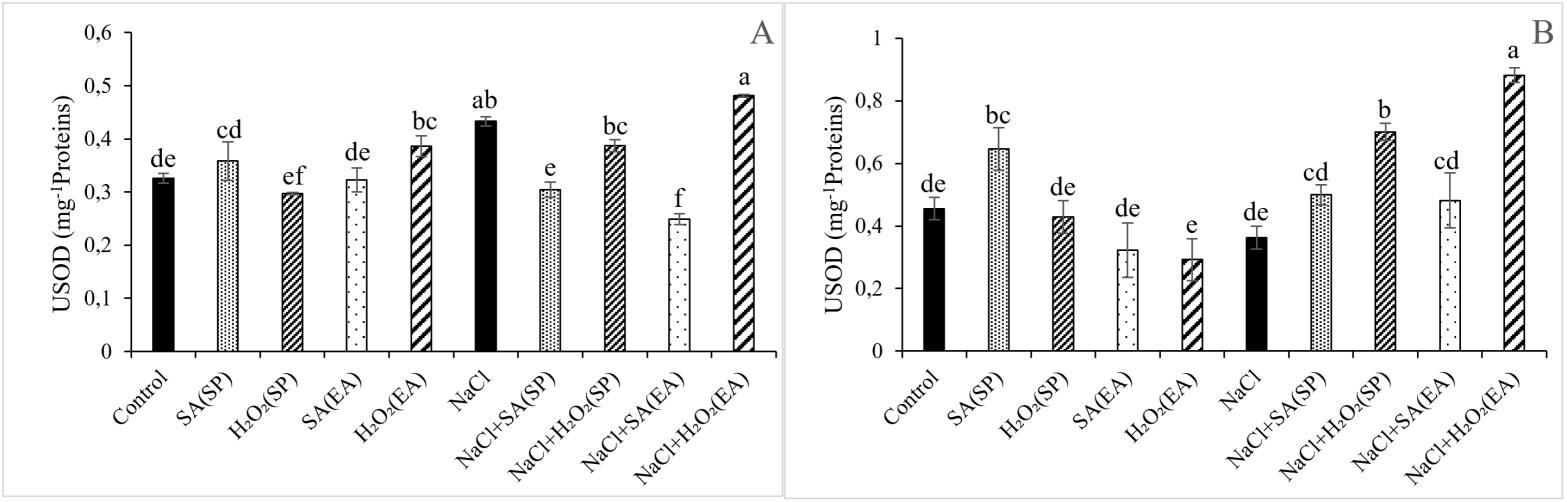
Effect of 0.1mM salicylic acid (SA) or hydrogen peroxide (H_2_O_2_) and salt treatment (0 and 75 mM NaCl) on the superoxide dismutase activity (SOD) in shoots (A) and roots (B) of lentil plants. SA and H_2_O_2_ are applied either by seed’s priming (SP) or added in the culture medium (EA). *Bars are means ± SE. Values with the same letter per parameter are not significantly different according to Duncan test (p = 0.05), n = 3.*

Data regarding the antioxidant enzymes activities SOD and GPOX under the different treatments are given in Figs 4 and 5. Except for root SOD activities for which no obvious effects were observed, salt stress remarkably increased shoot and root GPOX and shoot SOD activities, relative to controls. The effects of SA and H_2_O_2_ treatments on GPOX and SOD activities depended on the plant organ and on the method of application of the chemical agent. Irrespective of the method of their application, SA and H_2_O_2_ treatments sharply increased GPOX activities in roots of salt-stressed plants (by 130.02 and 125.89%, respectively). However, no obvious effects were observed in shoots. Similarly, shoot SOD activities were not affected by H_2_O_2_ treatment. Nevertheless, significant decreases were observed following SA treatment, whatever its method of application. All treatments noticeably increased root SOD activities, relative to those of salt-stressed plants, with the H_2_O_2_/SP and H_2_O_2_/EA treatments being more efficient (Fig 5).

## Discussion

Salinity is a major abiotic stress that negatively affects plant growth and development through the disruption of different physiological and biochemical mechanisms [6]. In this work, we observed significant reduction in biomass production of lentil plants by a 75 mM NaCl treatment (Fig. 1). Similar reductions in plant growth in response to salinity have been recorded in other plant species such as *Helianthus annuus* [45], *Hordeum vulgare* [46] and *Triticum durum* [47]. The observed reduction in lentil plants growth following salt application could be related to the nutritional imbalances, resulting in the reduction of several physiological activities [48]. Treatment of the salt-stressed lentil plants with SA and H_2_O_2_ recovered the plant growth and enhanced shoot and root DW (Fig. 1). This result on SA-ameliorating effects is consistent with those obtained in other legumes [49,50], wheat [51,52], sunflower [53], sugarcane [54] and tomato [55]. The application of H_2_O_2_ increased the DW of salt-stressed lentil plants, demonstrating its beneficial role in alleviating the negative effects of salinity. In pea plants, Dito et al. [56] attributed this amelioration effect of H_2_O_2_ to its action as a signaling molecule, and its involvement in the increase of the tolerance of plants to salinity through the regulation of various metabolic processes. It is worth pointing out that, in this work, the most prominent effects of SA and H_2_O_2_ on plant growth were obtained with the seed priming method, indicating the efficiency of this method in the mitigation of the adverse effects of salt stress on lentil growth. This could be linked to the induction of the antioxidant system (Figs 4 and 5), improvement of the membrane stability (Fig. 3) and amelioration of the gas exchange parameters (Table 2).

It is well known that salt stress disturbs ions uptake by plants, especially the K^+^ content witch adversely affects the K^+^/Na^+^ ratio in plant tissues [57,58]. As already noted for many plant species [59–62], our results showed that salt increases Na^+^ and decreases K^+^ contents in leaves and roots of lentil plants, and hence reduce the K^+^/Na^+^ ratio. SA and H_2_O_2_ applications, especially the H_2_O_2_ seed priming method, significantly decreased the Na^+^ and Cl^-^ contents and increased the K^+^ and Ca^2+^ contents in shoots and roots (Table 1). The involvement of SA application in the increase of nutrient uptake under salt stress has been indicated by several studies [18,63,64]. This may be explained by the protective effects of SA on cell membrane and root nutrient uptake systems [63]. Using H_2_O_2_ as seed pretreatment, Wahid et al. [65] showed that H_2_O_2_-treated wheat seedlings showed higher tissue K^+^, Ca^2+^, NO_3_^-^ and PO_4_^3-^ levels and induced K^+^/Na^+^ ratio. As K^+^/Na^+^ ratio in plants under salt stress is considered as one of the important selection criteria for salt tolerance [66], we can suggest that H_2_O_2_ treatment, particularly via the seed priming method, enhances the tolerance of lentil plants to salt stress. Likewise, the maintenance of Ca^2+^ transport under salinity constitutes another important indicator of salinity tolerance [67], making plants less sensitive to osmotic and ionic disturbances. In our study, this scenario seems to have occurred, especially following seed pretreatment with H_2_O_2_, since we observed significant increase in Ca^2+^ content in response to this treatment (Table 1).

As already mentioned for many plant species such as cotton [68] and sunflower [69], results of the current study indicated a marked reduction in all measured gas exchange parameters in plants subjected to salt stress (Table 2), reinforcing the deleterious effects of this constraint on lentil. The stomatal limitation caused by salinity affected the *gs* and thus reduced transpiration and Ci concentration. This could be linked to stomata closure and considered as a defense mechanism against water loss, which in turn decreases transpiration and CO_2_ assimilation [33,70,71]. SA and H_2_O_2_, regardless of the way of their application, mitigated the negative effects of salt stress on all measured gas exchange parameters. The beneficial effects of H_2_O_2_ on gas exchange parameters under salinity were reported in many plant species. For instance, Wahid et al. [65] indicated that treatment with H_2_O_2_ increased the photosynthetic capacity of salt-affected wheat seedlings through the improvement of the *gs* and the internal CO_2_ concentration. In bell pepper, Aragao et al. [30] observed that 15 µM H_2_O_2_ promoted the *gs*, CO_2_ assimilation, and carboxylation efficiency of salt stressed plants. It is worth pointing out that the beneficial effects of H_2_O_2_ on all gas exchange parameters depended on the method of application of this molecule, with the most prominent effects being obtained with the seed priming method. Similarly, the beneficial effects of SA on gas exchange parameters were reported in many plant species. Fariduddin et al. [72] found that, in *Brassica juncea*, photosynthetic parameters were induced following the exogenous application of SA. However, non-stomatal factors, such as the PSII efficiency, the Rubisco activity, and the ATP and NADPH production might also affect photosynthetic parameters under stressful conditions [73]. The observed ameliorated effects of SA on photosynthesis were related to induced effects on the pigment contents and Rubisco activity [74].

It is well known that photosynthetic pigments play an important role in light interception and energy transduction during photosynthesis [75]. As already indicated in several plant species [23,25,46,47], results of our study showed that salinity significantly reduced the concentrations of photosynthetic pigments in lentil plants (Table 3). The reduction of leaf chlorophyll under salinity could be due to increments of the chlorophyllase enzyme and to decline in chlorophyll biosynthesis [23]. In addition, during salinity stress, the high ROS production in cells causes oxidation and degradation of photosynthetic pigments [76]. Although SA and H_2_O_2_ significantly increased photosynthetic pigments of salt-stressed lentil plants, our findings showed that, as compared to the exogenous application, the seed priming method was more efficient in alleviating the negative effects of salinity on all photosynthetic pigment contents, especially following H_2_O_2_ application (Table 3). The reduction of the harmful effects of salinity following SA and H_2_O_2_ treatments could be achieved through the inhibition of chlorophyll oxidase enzymes, promotion of the activities of enzymes related to chlorophyll biosynthesis as well as decrease in the ROS contents and induction of the antioxidant defense systems [20,77].

In plants, MDA content serves as an indicator of membrane injury and physiological disorders [78]. Consistently with those in *Ocimum basilicum* [79], in Fennel [80], in wheat and barley [46,47], findings of this study showed that salinity provoked an oxidative stress indicated by the excessive generation of H_2_O_2_ and MDA (Figs 2 and 3), leading to a decline in the stability of the cell membranes [77]. Nevertheless, the application of 0.1 mM SA or H_2_O_2_ under saline conditions significantly decreased the H_2_O_2_ and MDA contents, especially in shoots with the seed priming treatment (Figs 2 and 3). The lower MDA and H_2_O_2_ contents observed in salt-stressed lentil plants following the application SA and H_2_O_2_ could be due to an efficient reduction in ROS damage and to a greater membrane protection, preventing thereby the unsaturated fatty acid damage and the electrolyte leakage [19,79]. It is now well documented that, to overcome the harmful effects of oxidative stress, plants activate the non-enzymatic and enzymatic antioxidant system [81]. POXs, CAT and SOD are the most important enzymes involved in oxidants detoxification [73]. Our study showed that salinity induced a significant increase in GPOX and SOD activities, especially in leaves, compared to control plants (Figs 4 and 5). This increase in antioxidants activities has been suggested as protection mechanism against salt-induced ROS as mentioned in cotton [82] and tomato [83]. The application of SA or H_2_O_2_ either as a seed pre-treatment or as an external application in the growing medium further stimulated GPOX and SOD activities. This result is in agreement with that published by Aazami et al. [3] in tomato; and seems to be directly linked to the reduction in MDA contents. In line with this, Xu et al. [19] and Shi et al. [84] showed that SA treatment mitigated the oxidative damage of *Saponaria officinalis* and *Cucumis sativa* seedlings, respectively by enhancing SOD, CAT and GPX activities, reducing thereby the lipid peroxidation levels compared to salt-stressed plants. Similar results were observed by AzevedoNeto et al. [85] in salt-stressed maize plants following the addition of H_2_O_2_ to the nutrient. As reported above, the enhancement of the antioxidant defense system in response to SA and H_2_O_2_ treatments has been reported in many crop plants under salt-affected conditions. Interestingly, results of this study showed that such an effect was more pronounced with the seed priming as compared to the root application method. The over activation of enzyme antioxidant system (Figs 4 and 5) and the subsequent reduction in ROS-induced membrane damage (Fig 3) in lentil plants following seed priming treatment are indication of the effectiveness of this treatment method in the mitigation in the salt-induced oxidative stress and, hence, in the amelioration of lentil plants growth under salt stress conditions.

In conclusion, our study demonstrated that salinity (i) decreases the growth and carbon assimilation in lentil plants, (ii) increased membrane alteration, lipid peroxidation and disrupted the uptake of essential ions. SA and H_2_O_2_ efficiently reduced the deleterious effects of salinity by inducing the growth and photosynthesis of lentil plants, by increasing the chlorophyll content and enhancing the plants antioxidant enzyme mechanisms, thus alleviating the membrane oxidative damage. Furthermore, our results indicate that the seed priming method, particularly with H_2_O_2_, is more efficient in alleviating the negative effects of salinity on lentil plants. This strategy could be recommended for obtaining better growth of lentil seedlings under salt stress. Therefore, the seed priming with H_2_O_2_ may be suggested as a potential strategy to alleviate the effects of salt stress in lentil plants.
